# Nongenetic Determinants of Age at Menarche: A Systematic Review

**DOI:** 10.1155/2014/371583

**Published:** 2014-06-23

**Authors:** Anna Yermachenko, Volodymyr Dvornyk

**Affiliations:** School of Biological Sciences, The University of Hong Kong, Pokfulam Road, Pokfulam, Hong Kong

## Abstract

*Background*. The acceleration of pubertal development is an important medical and social problem, as it may result in increased morbidity and mortality in later life. This systematic review summarizes relevant data about nongenetic factors, which contribute to age at menarche (AAM), and suggests those which may be the most important. *Methods*. The available literature from 1980 till July 2013 was searched using PubMed and Google Scholar databases. Finally, 154 papers were selected for the analysis. *Results*. Environmental factors, which may affect AAM, vary in populations of different ethnicity. The prenatal, infancy, and early childhood periods are the most susceptible to these factors. Body weight, high animal protein intake, family stressors (e.g., single parenting), and physical activity seem to influence AAM in most populations. *Conclusions*. The data about influence of nongenetic factors on AAM are still inconsistent. The factors affecting prenatal and early childhood growth seem to have a larger effect on further sexual maturation. Further studies are needed in order to validate the association between other environmental determinants and AAM in different ethnical groups.

## 1. Introduction

Menarche, a first menstrual bleeding, is a significant event in the reproductive life of a woman. Its onset is preceded by a complex cascade of hormonal changes during puberty. Endocrine regulation of sexual maturation is susceptible to various factors from the very beginning of prenatal life. The early onset of pubertal development is an important medical and social problem, as it may result in increased morbidity and mortality in later life [1]. Age at menarche (AAM) is one of the most significant traits, which is commonly used for retrospective epidemiological studies of female sexual maturation.

General improvement in socioeconomic conditions has resulted in the early onset of menses. Early AAM may be associated with health problems in later life such as breast cancer [2], type 2 diabetes [3], fertility impairment [4], cardiovascular diseases [5, 6], obesity [7], and psychological disorders [8, 9]. High risk of deviant behavior, early sexual debut, and smoking in adolescence, which are all related to early AAM, increase social importance of this trait [1]. Although there is evidence about an association between AAM and certain morbid conditions, its relationship with some unfavorable factors of prenatal development, childhood, and the prepubertal stage is still not clear.

There are two major groups of interacting factors, which can influence AAM-genetic and nongenetic determinants. The contribution of genetic factors to AAM is estimated to be about 57–82% [10–12]. Despite the apparently major role of genetic factors in AAM, environmental factors have gained increased attention, because many of them may potentially be controlled, which, in turn, may increase survival in later adulthood [13]. The role of some potential prenatal and postnatal determinants of AAM, such as maternal AAM, maternal weight gain during pregnancy, gestational diabetes, and birth size, has been studied during the last years [14–16]. However, a consensus about relative importance of these factors for AAM has yet to be reached.

This systematic review summarizes relevant data about nongenetic factors, which contribute to AAM, and suggests those which may be the most important.

## 2. Methods

**Figure 1:**
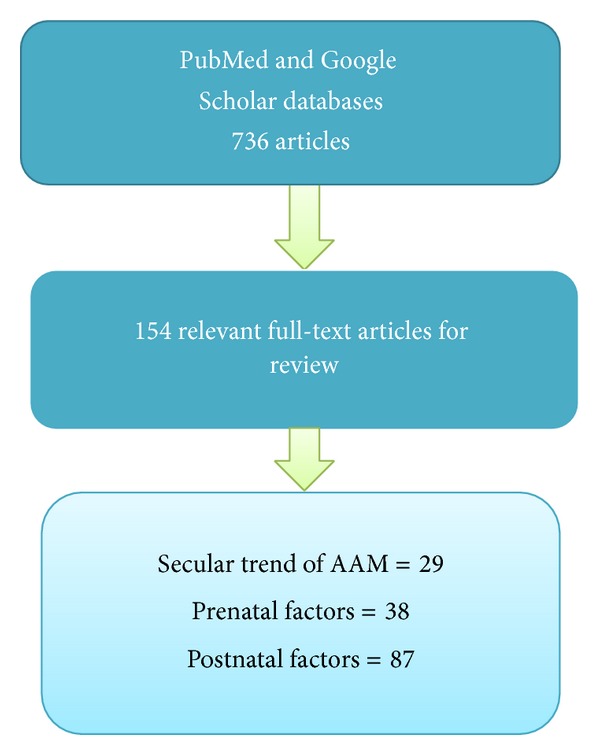
Flowchart of study selection for the systematic review.

The available literature from 1980 till September 2013 was searched using PubMed and Google Scholar databases. The following keywords were used in different combinations: “menarche,” “age at menarche,” “pubertal onset,” “menses onset,” “sexual maturation,” “pubertal growth,” “pubertal timing” with “ethnicity,” “birth weight,” “pregnancy,” “preeclampsia,” “prematurity,” “growth restriction,” “small gestational age,” “maternal weight gain,” “environmental hazards,” “endocrine disruptors,” “estrogen-like disruptors,” “maternal smoking,” “nutrition,” “diet,” “childhood obesity,” “adiposity,” “body weight,” “body composition,” “fat distribution,” “father absence,” “family composition,” “psychological disorders,” “socioeconomic level,” and “rural and urban residence.” This systematic review was conducted according to the guidelines of the PRISMA Statement [17]. The articles containing information only about premenarcheal stages of puberty and review articles were excluded. According to the above conditions, in total 736 articles were found and 154 articles contained data relevant to our study (Figure 1).

## 3. Results

### 3.1. Age at Menarche and Secular Trend in Populations of Different Ethnicities

The results of many studies indicate a downward trend of AAM in the main ethnic groups (Supplementary Table S1; see Supplementary Material available online at http://dx.doi.org/10.1155/2014/371583). The recent data for Caucasian females from various populations suggest that the current mean AAM varies from 11.96 to 12.93 [18, 19]. Females from the Southern European populations attain menarche slightly earlier than those from the northern part of the continent [18, 20]. Women of the European descent in the USA reach puberty around the same time as women in Europe. According to the US Census Bureau, Hispanics are considered as Caucasians [21]. Several studies reported that females of this ethnicity have lower AAM than whites and blacks [22, 23]. The downward secular trend in Caucasian populations seems to have stabilized in the last decades [24]. No significant differences in the secular trend between native Europeans and European descendants in the USA were found despite the significant genetic diversity of Caucasian Americans [22, 25].

The decrease in AAM is more significant in Asian women. Recent studies reported the mean AAM in Asians to be from 13 to 11.67 years [26, 27]. This is similar to Caucasians, but the secular trend is more pronounced in Asians. Chang and Chen [27] compared the onset of menarche in three generations of Taiwanese females and found it to be decreasing, on average, by one year per generation (15.16 ± 1.75, 14.50 ± 1.50, and 13.00 ± 1.26 years, resp.). Likewise, the difference between the minimum and maximum AAM within a generation reduced significantly just over three generations (i.e., from 10 years for grandmothers to 8 years for mothers and 7 years for daughters). At the same time, the proportion of Japanese girls who had AAM before 10 years increased from 0.0% to 2.1% among those who were born between 1930 and 1985 [28]. Two studies recently conducted in South Korea [29, 30] reported a significant drop of AAM (about 4 years) in women born between 1920 and 1986 that is nearly one year more than in Caucasian populations for the same period.

The data about AAM in black females were reported mainly from the United States [23, 31, 32]. Due to the significant genetic and environmental differences between African-American women and women from the African continent, it is difficult to compare the data about AAM in these populations [33]. The downward trend was observed in both populations but was associated with possible negative outcomes in adulthood only in African Americans [3]. The decrease in AAM in women from African countries is inversely associated with average body mass, which may reflect the socioeconomic growth and achievements in overcoming poverty. However, the data from African countries are quite limited to make a definite conclusion about the secular trend, although a decrease in AAM was reported [34].

### 3.2. Prenatal Factors Affecting AAM

#### 3.2.1. Maternal AAM

Since late 1980s, it has been supposed that prenatal environment may be associated with some pathological conditions in adolescence and adulthood [35, 36]. Recent studies [37–40] provided evidence for relationships between some anamnestic and clinical traits of mothers and AAM of their daughters. However, some controversy still exists [37, 41]. Females whose mothers had later AAM were reported to be at lower risk of early AAM [37]. It was found that maternal AAM may predict daughter's AAM in nonobese girls only; thus, high BMI in the prepubertal stage may modify some genetically predetermined traits [39]. Several morbid conditions related to high estrogen levels in mothers (marked by severe pregnancy-induced nausea) may be predictive for early AAM in daughters [37]. However, the results remain inconclusive due to the limited data available.

Some studies showed an association between AAM of mothers and obesity of their daughters [38]. In turn, obesity is a well-known risk factor of the early puberty onset. Early AAM of mothers was associated with early AAM and 3-fold higher risk of obesity in daughters [38]. Therefore, mother's AAM might be an indirect cause of the early onset of menses in their daughters.

#### 3.2.2. Maternal Weight Gain during Pregnancy

Endocrine dysfunction during pregnancy may affect hormonal status of a fetus [42]. Increased production of insulin during gestation results in excessive weight gain in the mother [43, 44], which may cause intrauterine growth restriction (IUGR) and premature puberty. Two possible prenatal mechanisms may contribute to this early puberty onset. Increased maternal body weight results in a high level of leptin, which is a factor for fetal growth retardation and intrauterine insulin resistance [45]. The excessive fat in a mother may decrease insulin sensitivity in a fetus and lead to hyperglycemia that induces pancreatic *β*-cell proliferation [40, 46]. As a consequence, these cells produce more insulin that may result in hyperinsulinemia in the fetus. There is a U-shape association between gestational weight gain and early AAM of daughters [40]. Both extremes of weight gain in pregnant women, that is, low (less than 10 lbs.) and high (more than 40 lbs.), may be considered as risk factor for earlier AAM in daughters.

#### 3.2.3. Prenatal Exposure of Preeclampsia

Preeclampsia is one of the most common causes of IUGR. Low birth weight is associated with impaired metabolic status after birth resulting in catch-up/early pubertal growth. Therefore, preeclampsia potentially may contribute to the early onset of puberty. However, there is no clear evidence about an association of this pregnancy complication with AAM [15, 47, 48]. Some researchers [48, 49] found only a slight decrease in AAM in daughters of women with preeclampsia. Univariate analysis of recent data suggests that exposure to low endogenous estrogens* in utero* during preeclampsia might decrease AAM. However, the multivariate analysis did not corroborate these results [15].

#### 3.2.4. Prematurity

Preterm birth is one of the most common complications of pregnancy and may potentially affect metabolic status in childhood and puberty [35]. However, recent studies did not determine any significant association between prematurity and AAM [35, 50, 51]. Szwed and Kosińska [51] demonstrated a considerable variation of AAM in Polish girls born before 37 weeks of gestation. Moreover, the later onset of menarche was observed in premature girls with weight below 2,500 g. The same trend for the onset of puberty was found in Asian adolescent girls [50]. The girls born prematurely had 4 months delay in the onset of sexual maturation as compared with those who were born in or after 41 gestational weeks (time ratio =0.41; 95% CI 1.01–1.06).

#### 3.2.5. Environmental Hazards Exposure during Pregnancy

Intrauterine exposure to some endocrine disruptors during pregnancy may lead to premature puberty and, as a result, to early AAM of the offspring [52–57] (Supplementary Table S2).

A general consensus is that exposure to environmental hazards during gestation has more dramatic consequences for the offspring than that during breastfeeding, early childhood, or puberty. The most widespread xenobiotics are halogen-containing organic compounds (dichlorodiphenyltrichloroethane (DDT)/dichlorodiphenyltrichloroethylene (DDE), polychlorinated biphenyls (PCBs), polybrominated biphenyls (PBBs), bisphenol A, phthalates, and dioxins) and heavy metals (lead and mercury) [42, 57, 58]. Organochlorine compounds may disrupt human pregnancy by affecting gametogenesis, fertilization, implantation, and early development of a conceptus. Females exposed to PCBs during antenatal development may have lower fecundity [59]. Accidental PBB exposure through contaminated dairy and meat products and premature pubarche with normal thelarche onset [55]. Supposedly, the exposure to halogenated organics in the prenatal period might disrupt endocrine regulation of puberty timing. On the other hand, another study reported no association between the high dose exposure to another estrogen disruptor, dioxin, and sexual maturity disorders in premenarcheal girls [53]. Despite that, studies on animal models suggest that fetuses might be more susceptible to such doses of dioxin (e.g., [60]).

The population of fish eaters consuming fish contaminated by DDE and PBCs did not show a significant effect of the halogen-containing substances on AAM. One study has shown that consumption of contaminated fish can result in DDE levels up to 15 *μ*g/L in serum and that it can decrease AAM by 1 year (−0.07 years per *μ*g/L concentration) [61]. No such effect of PBC was found.

The observations of the prenatal exposure to halogen-containing compounds suggested that the pregnant women likely were exposed to a mixture of chemicals with estrogenic, antiestrogenic, and antiandrogenic action, which might have opposite effects on the endocrine system [62]. Another study provided evidence that* in utero* exposure to xenobiotics resulted in changing in puberty onset of the offspring, while postnatal exposure was related to the acceleration of the process of sexual maturation [54].

Other types of exogenous hazards for female fetuses are substances consciously consumed by pregnant women, such as tobacco, alcohol, and drugs. These products are potential endocrine disruptors and may affect sexual maturity of the female offspring in the postnatal period. One of the well-studied factors is maternal smoking [63–65]. There is evidence that mother's smoking can lead to obesity and early sexual maturation of her female offspring [40]. The deleterious effect of tobacco leading to fetal growth restriction was attributed to accumulation of cadmium in human placenta [66]. Cadmium decreases production of progesterone due to disruption of leptin synthesis [67, 68]. Nonwhite American girls prenatally exposed to high doses of nicotine had about 6-month earlier menarche onset [65]. However, later the same authors reported differently [64], probably due to several other prenatal factors related to AAM, which were included in the analysis. They found that daughters of women who smoked heavily (more than 20 cigarettes per day) during gestation had mean AAM about 3.7 months later. These findings were supported by another study [63] that showed a 5.3-month delay in timing of menarche in daughters whose mothers were heavy smokers during pregnancy. A possible mechanism of this delay is a toxic effect of polycyclic aromatic hydrocarbons of cigarette smoke on embryonic ovaries. However, the consensus is still absent: the most recent study [69] reported that daughters of heavy smoking women (more than 10 cigarettes per day) attained puberty 6.5 months earlier.

Alcohol consumption during pregnancy is a known cause of IUGR that might be pathogenically associated with early AAM. However, the association between prenatal exposure to alcohol and AAM was not found [65]. Even in cases with fetal alcohol syndrome, AAM was not affected [70].

#### 3.2.6. Birth Weight

Birth weight is one of the significant factors related to AAM, but biological mechanisms of this relationship are still unclear [51, 71]. Higher birth weight, as well as lower weight for gestational age, may contribute to the early onset of puberty [72]. However, by and large, the data about an association between birth weight and AAM are inconsistent [37, 73–75] (Supplementary Table S3).

IUGR is a condition, which may lead to premature pubarche, polycystic ovary syndrome, and infertility [35]. The experimental study in rats with IUGR induced by uterine arteries ligation showed a delay in puberty, probably due to the insufficient body mass at the time of sexual maturity [76]. Studies on human reported the contrasting results. Girls born small for gestational age (SGA) had an earlier stage of thelarche than those who were appropriate for gestational age (AGA). The SGA and AGA girls had no significant difference in BMI and levels of androgens in the prepubertal stage [35]. This generally supports the hypothesis that SGA leads to premature puberty [77]. The data from 1,000 Philippine girls [78] showed that the girls who were longer (more than 49 cm) but thinner (less than 3 kg) at birth reached menarcheal age 6 months earlier than those who had been shorter (less than 49 cm) at the same gestational age. The author suggested that maternal nutritional status during pregnancy and breastfeeding and some socioeconomic parameters should be taken into account when discussing AAM in low-income countries.

The results from Caucasian females support the association between small size at birth and AAM [71]. The possible mechanism might be related to the high serum level of androgen, dehydroepiandrosterone sulphate (DHEAS) during puberty, which was inversely correlated with the size at birth. The high level of DHEAS in SGA children is probably related to early sexual maturity and could affect adrenal androgen production in adulthood [71].

### 3.3. Postnatal Factors Affecting AAM

#### 3.3.1. Consumption of Alcohol, Tobacco, and Drugs and AAM

Consumption of alcohol, tobacco, and drugs before adolescence is generally uncommon and, therefore, the data about their possible contribution to AAM are limited and the results are inconclusive (Supplementary Table S4). Only few studies examined prepubertal smoking and further sexual maturation. The most recent study of a large cohort of girls did not show any associations between AAM and active consumption of various drugs (e.g., marijuana and cannabis products, hallucinogens, glue, solvents, etc.) in the premenarcheal and menarcheal period [79]. The earlier study, which included 59 active smokers at pubertal age before menarche [65], reported a positive association with later AAM. The authors suggested that this was related to more opportunities for girls with later AAM to start smoking earlier.

Passive smoking is more common and has been studied as a postnatal factor affecting sexual maturation. However, the results are also inconsistent. An early report [80] showed the relationship of smoking with early AAM in a large cohort of Polish girls. The association between environmental smoke exposure and early AAM was weak [65]. Conversely, another study [63] reported an association between passive smoking during childhood and later AAM. The authors suggested that these inconsistencies may be due to the different proportions of white and nonwhite girls in the studied cohorts who have significant differences in socioeconomic status (SES) [63].

#### 3.3.2. Body Weight, Fat Distribution, and AAM

The Frisch-Revelle hypothesis [81], which states that the onset of menses is positively associated with increasing body fat in pubertal girls (22% or 48 kg of body weight, approximately), is somewhat outdated, especially with respect to the definition of “critical weight.” However, despite some skeptical remarks and critical comments from the 1970s [82–86] until now, the Frisch-Revelle hypothesis proposes reasonable explanations of how body fat affects menarcheal onset. The identification of the leptin gene [87] gave further support for the association between excessive body fat and early sexual maturation at the molecular level. Leptin stimulates pulsatile release of gonadotropin releasing hormone in the hypothalamus that serves as a signal for the onset of menarche [88, 89].

Basically, there are two weight-related factors associated with AAM: total body weight measured as body mass index (BMI) and various measurements of fat distribution (Supplementary Table S5). The majority of the studies corroborated the association between high BMI in infancy, prepuberty, puberty, and earlier AAM. Fat distribution was a focus of several studies, but the data about its influence on AAM are inconsistent. Guo and Ji [7] reported a strong association between large waist circumference and early AAM. They emphasized that this parameter has more predictive value than body weight for possible adverse outcomes in adulthood. Other data suggested that gluteofemoral fat distribution had a larger effect on the onset of menses than upper body fat and overall body fat [90]. Lower body fat may produce more leptin than adipose tissue of the other parts of the body; each centimeter of the hip circumference can double the leptin levels as compared with the waist circumference [90]. On the other hand, some studies did not find an association between fat distribution and the timing of puberty events [91].

Numerous studies suggest that postnatal weight gain is a result of prenatal fetal programming [92–94]. High BMI may decrease AAM. In most cases, the effect of the excessive postnatal body weight on early AAM is a consequence of IUGR or SGA [75]. The time of postnatal weight catch-up is a very important predictive factor for AAM [15]. The earlier postnatal weight gain has a stronger association with earlier AAM that usually occurs in children with IUGR [95]. Therefore, it is very important to study association between not only AAM and postnatal weight gain, but also other cofactors from the antenatal anamnesis.

#### 3.3.3. Nutrition (Incl. Breastfeeding) and AAM

Diet in infancy and early childhood is important for reproductive health. There is evidence that breastfeeding can prevent excessive weight in childhood [96, 97]. Given the possible association between obesity and the onset of puberty, it is likely that breastfeeding may decrease the risk of early puberty. However, recent studies on a Hong Kong population [98, 99] did not find any association between milk consumption, duration of breastfeeding (more than 3 months), and the timing of puberty in Chinese children. Likewise, no association between the duration of breastfeeding and AAM was determined in a cohort of Philippine girls [100]. On the other hand, formula feeding during early infancy might promote weight gain and earlier AAM in Asians and Caucasians [95, 101]. These results gained further support from a study using a rat model, which showed that feeding enriched protein formula has more deleterious effect on IUGR rats than on the animals with normal birth weight due to rapid weight gain in early life [102]. As mentioned above, IUGR is one of the major risks of early AAM. The excessive protein intake from formula feeding in early life may potentiate this adverse effect.

There are three major categories of dietary products and supplements, which may influence the timing of female sexual maturation: those containing animal fat or protein [103–106], soybean products [107, 108], and the fortified products with supplements like dietary fiber, calcium, and vitamin D [106, 108, 109] (Supplementary Table S6).

Cow milk seems to be the most commonly used animal product consumed by girls before attaining menarche. Very high protein, calcium, and mineral content of cow milk may be a trigger of growth in early infancy [110]. Animal protein can stimulate insulin growth factor 1 (IGF-1), a principal regulator of growth in humans [111]. This effect of cow milk consumption on sexual maturation is most noticeable only in the periods of active growth, for example, in early childhood and adolescence [103, 110].

In recent years, soybean products for children became increasingly popular even in regions where soy is not a part of a traditional diet [107]. Some soy isoflavones are known to act as mild endocrine disruptors [112]. Isoflavones can cross the placenta and be secreted in breast milk. The most intensive consumption of soy-containing products in early childhood occurs during formula feeding. Depending on their concentration, isoflavones may either induce or repress expression of gonadotropin releasing hormone (GnRH) [112].

Vitamin D deficiency may indirectly contribute to the early onset of puberty via childhood obesity.

Dietary fiber is a group of heterogeneous products, which may prevent early AAM through their effect on estrogen metabolism [105]. However, the data about this effect are controversial and its mechanism is still unknown [108, 113]. Dietary fiber may reduce estrogen reabsorption and deconjugation in the gut and thus protect against excessive estrogen supply [114].

#### 3.3.4. Environmental Hazards

High exposure to some endocrine disrupting chemicals (EDCs) in childhood may lead to rapid sexual maturation. Although the number of known EDCs is quite large, little is known about their impact on puberty [114]. In recent years, mainly estrogen-like EDCs (organochlorine chemicals) and heavy metals (lead and mercury) have been the research focus as possible factors altering female sexual maturation (Supplementary Table S7). Wide distribution of plastic products increases exposure to various organochlorine compounds (PBBs, PCBs, DDT/DDE, dioxin, etc.) and thus makes them possible contributors to AAM [115]. This group of chemicals can affect the endocrine system in various ways: through estrogenic, antiandrogenic, and steroidogenic enzymes, neurotransmitter receptors, and the others [116]. However, the evidence about a possible effect of organochlorine chemicals on AAM is inconclusive. For example, while the studies on animal models reported delays in attaining puberty caused by an exposure to organochlorine compounds, such effect was seldom observed in humans [53, 55, 117]. On the other hand, there is evidence that exposure to DDT/DDE may lower AAM [118, 119]. This inconsistency may stem from the fact that humans are usually exposed to multiple EDC, which makes it difficult to estimate an effect of any particular chemical on AAM. On the other hand, different chemicals may augment the adverse effect through a synergistic action even in low concentrations [58].

Lead is one of the most widespread heavy metals, which is present in paints, lead pipes, industrial waste, and lead-containing petrol. It can affect female growth in puberty by inhibiting IGF-1 [120]. Lead in low doses may be associated with the earlier onset of menses, whereas the exposure to its high levels may delay AAM [58, 120].

Some personal care chemicals, such as hair care products, used every day during childhood can lower AAM [121]. However, the results are still inconclusive due to the limited data available [122].

#### 3.3.5. Psychological Factors and Their Impact on AAM

Since 1991, when the evolutionary theory of socialization was published [123], the psychosocial factors, which can influence female sexual maturation, have gained increased attention of scientists. Family disruption, childhood adversity, and continuous stress may accelerate sexual and reproductive development of girls. Risky behavior, adolescent pregnancy, drug addiction, and early first sexual intercourse may be consequences of family disharmony, especially if it happens during the first 5–7 years of life. Single parenting and stressful situation in a family may speed up reproductive maturity of girls [124]. Another hypothesis suggests that male carriers of the X-linked androgen receptor gene may be predisposed to family disruption due to the short allele GGC repeat polymorphism, which is associated with destructive behavior and aggression [125]. Their female offspring inheriting this polymorphism is predisposed to precocious puberty. This study provides a genetic basis for Belsky's theory that states that individuals are affected in varying degrees depending upon their early childhood experiences and the qualities of the environment [123]. On the other hand, some studies did not find any association between the androgen receptor polymorphism and male adverse behavior [126].

Father's absence is one of the most studied psychological factors, which is significantly associated with early puberty of the offspring [127–133] (Supplementary Table S8). Family desertion by a father significantly contributes to early puberty of a child, especially if it happened in early childhood (before 5 years of age). Later separation of parents (i.e., during the prepubertal period of a child) may lead to the risky behavior of an adolescent, such as early sexual contacts and numerous sexual partners, but not to the earlier onset of menses [127]. In disrupted families, younger sisters attain menarche earlier than the elder ones, because they spend less time with their fathers [129]. Girls at the age between 0 and 5 years seem to be the most sensitive to family composition [130]. The presence of a stepfather in a family at girl's age of 10–15 may influence AAM of a stepdaughter [127]. Belsky et al. [123] suggested that the absence of a father may result in metabolism slowdown, subsequent increased fat accumulation in daughters, and, respectively, early menarche. However, Matchock and Susman [131] did not find any differences in the incidence of obesity in daughters between one-parent and two-parent families.

Another psychological factor is adverse childhood experience, such as sexual abuse [126, 134–137]. Life problems in childhood like an alcohol addicted father, mother with nervous troubles, and sexual abuse are strongly associated with early sexual maturation [126, 137]. Physical abuse can also speed up pubertal development [136].

In addition to family problems, psychological disorders in early childhood can also affect the onset of menses [138–140]. High levels of androgens during the prenatal period were reported as a possible risk factor for autistic-like disorders and later onset of puberty [139], but this finding remains under question [140]. All psychological conditions leading to malnutrition, such as anorexia nervosa [141], affect AAM due to low leptin levels that results in impaired secretion of GnRH [142].

#### 3.3.6. Physical Activity and AAM

AAM delay is well documented for females exposed to regular high intensive physical exercises in childhood and adolescence [142–146]. Abnormalities of menstrual function occur in 6–73% of female athletes [145]. Some physical activities (e.g., ballet) in combination with nutritional deficiency may influence the onset of puberty [147] and lead to, on average, 1-year delay in AAM in Caucasians [144].

#### 3.3.7. Socioeconomic Factors and AAM

Socioeconomic factors have a significant impact on nutritional and psychosocial status during childhood and adolescence and may influence AAM (Supplementary Table S9). These factors include type of residency, parental educational and occupational level, family size, household income, and immigration status. The majority of studies reporting the association between AAM and socioeconomic status (SES) were conducted in developing countries [148–153]. All these studies compared the influence of SES on AAM in poor rural regions and in more developed urban areas. Lower AAM is considered as a positive trend reflecting better socioeconomic conditions and welfare in the countries where famine, drought, and local conflicts are still serious problems. Only one study conducted in Nigeria did not show significant differences between AAM in rural and urban areas probably due to similar living conditions in the rural areas and the cities [149].

In upper-middle income countries, the impact of SES on AAM is still significant [152, 154–158]. Better SES led to a decrease in AAM of urban adolescent girls as compared to their rural counterparts. However, several studies did not report any association of AAM with socioeconomic factors [152, 159, 160]. The inconsistency might be due to some unknown environmental factors, which might influence AAM [154].

In developed countries, socioeconomic factors apparently do not play a meaningful role in the onset of puberty [20, 160, 161]. Nonetheless, low SES was associated with earlier AAM in African Americans and Hispanics in the USA [162, 163].

## 4. Discussion

The interest in the environmental factors that influence the onset of puberty has significantly increased during the last three decades. However, despite extensive studies, the mechanisms by which the environment influences the onset of menarche remain largely unclear.

### 4.1. Ethnic Background

Genetic and lifestyle factors affecting AAM vary in populations of different ethnicity. In general, although all major ethnic populations have the downward secular trend of AAM, interethnic rates of these changes are different. There is evidence that ethnic background may be an important factor contributing to the prevalence or expression of various traits in populations [164, 165]. These interethnic differences are associated primarily with respective differences in the genetic structure of populations [166]. On the other hand, in the case of lifestyle factors (e.g., diet, family relationships, etc.), the differences in their effect on AAM may be largely attributed to the different cultural background.

### 4.2. Prenatal Factors Affecting AAM

#### 4.2.1. Mother's Traits

Despite the large number of studies of mother-daughter pairs, no significant association was found between maternal characteristics (AAM, pregnancy complications, and weight gain during pregnancy) and AAM in daughters. The apparently best approach to identify the association between mother's traits and daughter's AAM is prospective longitudinal cohort studies involving both mothers and daughters. However, this type of study is hardly doable due to time and financial constraints. Another possible cause of the abovementioned inconsistency is mother's AAM recall bias [167]. Furthermore, the secular trend increases the gap between mother's AAM and daughter's AAM and thus contributes to the bias too. One of the possible confounders that may influence the association between mother's AAM and daughter's AAM is BMI in prepubertal stage [39].

#### 4.2.2. Prenatal Hazard Exposure

Exposure to environmental hazards during prenatal period of life may be a result of anthropogenic pollution or conscious consumption of some toxic substances by a pregnant woman. However, it is quite difficult to determine an effect of a particular toxin on AAM specifically in a prenatal period due to possible further exposure during breastfeeding and early childhood.

Tobacco smoking during pregnancy is probably the most common life habit affecting a foetus* in utero* [63–65, 168, 169]. However, the data about its effect on AAM are inconsistent. As mentioned above, they may be confounded by the postnatal exposure. A number of other important factors should also be taken into account: the timing of tobacco exposure during pregnancy and whether mother only or both parents are the active smokers. Susceptibility of a foetus to tobacco is different for each trimester and increases to the end of gestation [19]. Polycyclic aromatic hydrocarbons affect the sperm cells of smoking fathers that may lead to the developmental impairment during early embryogenesis [170].

#### 4.2.3. Birth Weight

Insulin resistance, which leads to the rapid growth catch-up and further excessive weight gain in SGA born children [171], may suggest an association between early sexual maturation and SGA. The low birth weight may result from three different conditions: IUGR, when the foetus cannot achieve its genetically determined body size parameters due to some pathological process leading to chronic hypoxia; SGA, including all cases of IUGR and those normal foetuses that have genetically determined small weight; and prematurity. These three conditions utilize different pathways and therefore may have different implications in AAM. Despite that, in most studies, the low birth weight (<2,500 g) is considered as a single phenotype, which may introduce a bias to the results. Another potential cause of the bias is that different studies account for different confounding factors. That is why the results of studies utilizing the univariate linear regression analysis are different from those employing the multivariate analysis [78]. Some researchers emphasize that body weight should be analysed in conjunction with growth pattern in early childhood in order to find the association with the onset of menses [172].

### 4.3. Postnatal Factors Affecting AAM

#### 4.3.1. BMI

The Frisch-Revelle hypothesis essentially states that the increase of BMI in childhood and adolescence is the most reasonable explanation of early AAM [81]. Excessive weight in early puberty is associated with a high risk of being overweight in adulthood. It was found that obesity-associated loci produce a larger effect in adolescents than in adults [173]. Moreover, ethnical factors can modulate the response of these loci on the weight gain [174]. In fact, Asians have a higher risk for obesity and obesity-related conditions than Caucasians [175]. Insulin resistance that drives obesity has a genetic basis, but ethnicity plays a significant role in the phenotypic expression [176].

However, although average BMI of Asians is lower than that in Caucasians, the decrease in AAM is more pronounced in Asians. Supposedly, despite lower BMI, Asians have more body fat, mainly visceral, than Caucasians [177], and, therefore, BMI alone is probably not a good indicator for the risk of early AAM. The most recent study [165] proposed to adopt a lower threshold BMI value for classification of overweight and obesity in Asians. Therefore, new data are needed to revise the BMI levels, which may potentially affect AAM in Asians.

The association between early onset of menses and increased body weight is apparently related to the ability of leptin to stimulate pulsatile secretion of GnRH. Having been produced by fat tissue, leptin communicates to the hypothalamus that critical amount of fat (nearly 16 kg) has been accumulated and the late stage of puberty can start. The fact that AAM has negative correlation with leptin concentration is evidence in support of the Frisch-Revelle hypothesis [89].

Despite the data about the association between high BMI and early AAM [171, 178, 179], some questions remain unanswered. For example, at what age is the body more vulnerable to fat accumulation, early childhood or prepubertal stage? Which anthropometric characteristics may increase predictive value of BMI for AAM? What is more important for AAM, upper or lower body fat? As mentioned above, body composition is an ethnic-dependent trait and is quite different in Asians, Caucasians, and Africans. Given that, should the BMI scale be the same for all ethnical cohorts?

#### 4.3.2. Nutrition

The association between alimentary habits and the tempo of child growth suggests that some nutrients may potentially accelerate the timing of menarche [169]. Feeding with formula (especially soy-containing) during infancy may decrease AAM [109]. It is still unclear to what extent the duration of formula feeding and various ingredients of the formula may affect AAM. On the one hand, consuming animal protein (cow milk and meat) and soy-containing products may promote early AAM through the excessive energy intake and subsequent overweight or estrogen-like action that accelerates pubertal development. On the other hand, other common nutrients, such as vegetable protein, fibres, and vitamin D, may act protectively against the rapid sexual maturation. The availability of the large number of high-fibre and vitamin D fortified products on the market prompts further studies to understand their potential impact on AAM.

#### 4.3.3. Postnatal Environmental Hazard Exposure

Most studies about the environmental hazard exposure did not show its association with onset of menarche [53, 55, 117]. The number of reports was focused on EDCs. Studies of residual levels of several organochlorine chemicals in serum showed negative correlation with AAM [119, 180]. Studies of the prenatal exposure to environmental hazards are more biased than those of the postnatal exposure. The most serious limitation is the time between the prenatal EDCs exposure and AAM. Generally, the mean levels of EDCs in the cohorts of pregnant women are unknown. The capability to cross the placenta is unclear for most EDCs. Even in cases when this capability was determined, concentrations of EDCs that can reach and affect the foetus are not known. The same problems are about contamination of breast milk by organochlorine compounds. One of the possible ways to clarify the role of EDCs in pubertal development is measuring their levels in the body fluids at the moment of exposure during prepuberty and puberty.

#### 4.3.4. Psychological Disorders

In general, family stressors decrease AAM. Absence of a biological father is the most common psychological problem leading to the early onset of menses [129, 130]. Influence of a stepfather and male siblings on AAM of stepdaughters is not clear. The most vulnerable age for family stressor exposure, such as parental separation, is up to 7 years [127]. Most findings support an association between stress in early life and early AAM. However, this factor should be considered along with the other determinants, such as BMI and socioeconomic status [181].

#### 4.3.5. Physical Activity

There is a consensus that excessive physical activity delays AAM, whereas the moderate and light physical training do not affect AAM. The mechanism of delayed puberty in adolescent athletes is probably related to the decreased leptin release resulting in primary hypothalamic amenorrhea at least in 50% of female adolescent athletes [142].

#### 4.3.6. Socioeconomic Factors

Nowadays, socioeconomic factors apparently become less significant for AAM as compared to the last centuries when consistent nutritional deficit was a major problem worldwide [20]. However, these factors are still important for low-income countries. According to the paradigm for research on SES and health [182], SES is one of the main determinants that can affect human health. In developing countries, a decrease in AAM usually reflects the improvement of the nutritional status of adolescents [183]. In contrast, in the well-off countries, early AAM is associated with high prevalence of obesity among low-income people [32]. Therefore, this difference between developed and developing countries may explain the inverse association between SES and body mass and other lifestyle factors, such as nutrition and physical activity [162].

The data about the influence of environmental factors on AAM may be summarized using the SWOT (strengths, weaknesses, opportunities, and threats) approach (Table 1), which is widely used in public health studies (e.g., [184]).

## 5. Conclusions

The data about influence of nongenetic factors on AAM are still largely inconclusive. Factors affecting prenatal and early childhood growth have more serious consequences for sexual maturation than those affecting adolescence. Body weight, high animal protein intake, and single parenting are among those which are likely associated with early AAM. Further investigations are needed to confirm the association between environmental determinants and AAM in different racial/ethnical groups and to resolve the existing inconsistencies.

## Supplementary Material

Original articles focused on AAM as primary or secondary outcome were extracted from PubMed and Google Scholar databases. All studies were classified and organized in the tables depending on time (prenatal or postnatal) and type of environmental exposure. Information about mean AAM and secular trend in different countries and ethnic groups was selected from the most recent articles only. Mean AAM and difference in AAM means between exposed and nonexposed groups was given in years. Association between different exposure and AAM was considered as significant, if p<0.05.

## Figures and Tables

**Table 1:** Strengths, weaknesses, opportunities, and threats for studies on the nongenetic factors affecting AAM.

Strengths	Weaknesses
(1) The large number of studies for the last two decades identified many potential environmental determinants of AAM (2) Large volume of data on physiology of puberty and reproduction is available (3) Advanced bioinformatics methods and powerful computer technologies make it possible to analyse large data set of data (4) Critical mass in reproductive epidemiology, physiology, and endocrinology	(1) Aetiology of menarche is largely unknown (2) The results of different studies are often inconsistent (3) Data from non-Caucasian populations are limited (4) It is difficult to determine the effect of any single factor (5) There are many potentially important but yet unknown factors, which may confound the results (6) Data about gene-environment interactions and their effect on AAM are scarce (7) Persistent migration processes worldwide make it difficult to recruit ethnically homogenous cohorts (8) Continuous variation of many environmental factors makes it difficult to determine the effect size (9) Animal models are often inappropriate for studying an effect of many environmental factors on AAM

Opportunities	Threats

(1) Use homogenous ethnic populations for epidemiological studies of AAM (2) More emphasis on the exposure to the environmental factors during prenatal and early childhood period, during which their effect is more pronounced (3) Collaborative efforts of research groups from different countries will help to increase efficiency of the epidemiological studies of AAM (4) Implementation of the results in preventive medicine and public health management.	(1) Potential exposure to new factors (e.g., chemicals) with an unknown effect on the female reproductive system (2) The number of environmental factors affecting AAM may be very large (3) Several generations are often needed to determine a consistent cause-effect relationship between environmental determinants and AAM (4) Epidemiological studies in the low-income countries are difficult to conduct due to the lack of sufficient funding (5) Methodology of the result application in public health management is still not well developed
