# Supplementary material for: Nongenetic Determinants of Age at Menarche: A Systematic Review
Source: Biomed Res Int. 2014 Jun 23;2014:371583. doi: 10.1155/2014/371583 (PMC4094877; doi:10.1155/2014/371583)
Supplement: Supplementary file 1 — Original articles focused on AAM as primary or secondary outcome were extracted from PubMed and Google Scholar databases. All studies were classified and organized in the tables depending on time (prenatal or postnatal) and type of environmental exposure. Information about mean AAM and secular trend in different countries and ethnic groups was selected from the most recent articles only. Mean AAM and difference in AAM means between exposed and nonexposed groups was given in years. Association between different exposure and AAM was considered as significant, if p<0.05. [file 371583.f1.pdf]

Table S1: Mean age at menarche in different populations

Countries	Mean AAM, years	Secular trend	References
CAUCASIANS			
**France**	12.00	n/a	[23]
**Italy**	12.40	From 14.1 to 12.4 (survey in 1930 and 2000)	[20]
**Croatia**	12.31	From 12.82 to 12.31 (survey in 1990 and 2010)	[186]
**Spain**	12.83	From 13.72 to 12.83 (born in 1925-29 and in 1958-62)	[187]
**United Kingdom**	12.93	n/a	[19]
	12.30	From 13.5 to 12.3 (born in 1908-19 and in 1990-93)	[25]
**Ireland**	12.53	From 13.52 to 12.53 (survey in 1986 and 2006)	[188]
**Bulgaria**	11.96	From 15.0 to 11.96 (survey in 1904-06 and 1994-2000)	[18]
**Poland**	12.73	n/a	[189]
**USA**			
White Americans	12.50	From 13.3 to 12.5 (born before 1920 and after 1980)	[22]
Hispanics	12.20	From 13.2 to 12.2 (born before 1920 and after 1980)	
White Americans	12.57	n/a	[23]
Hispanics	12.24	n/a	
White Americans	12.55	n/a	[31]

Hispanics	12.25	n/a	
White Americans	12.50	n/a	[32]
Hispanics	11.54	n/a	[129]
White Americans	11.50	From 12.3 to 11.5 (1978-1979 and 1992-1994 two cohorts study)	[190]
ASIANS			
**China**	12.80 (urban area)<br>13.20 (rural area)	n/a	[157]
average	12.76	n/a	[161]
urban	12.60	From 12.88 to 12.60 (survey in 1995 and 2005)	
rural	12.92	From 13.26 to 12.92 (survey in 1995 and 2005)	
	12.43	n/a	[155]
	11.67	n/a	[26]
	13.46*	n/a	[191]
**Japan**	12.20	From 13.8 to 12.2 (born in 1930-1980)	[28]
**South Korea**	13.10	From 16,90 to 13,10 (born in 1920-1986)	[29]
	12.70	From 16.8 to 12.7 (born in 1920-1986)	[31]
**Indonesia**	12.96	n/a	[192]
**Hong Kong**	12.30	n/a	[193]

**Taiwan**	13.00	n/a	[27]
**Nepal**	12.69	n/a	[194]
AFRICANS			
**Ghana**	13.98	n/a	[195]
**Mozambique**	13.90	n/a	[196]
**Gambia**	14.90*	From 16.06 to 14.90 (survey in 1989-2008)	[34]
**Mali**	14.72	n/a	[34]
**Senegal**	15.90	n/a	[34]
**Ethiopia**	15.80	n/a	[152]
**USA**			
Afro-Americans	12.20	From 13.60 to 12.20 (born before 1920 and after 1980)	[22]
Afro-Americans	12.52	n/a	[23]
Afro-Americans	12.06	n/a	[31]
Afro-Americans	12.00	n/a	[32]
Afro-Americans	11.40	From 12.3 to 11.4 (1978-1979 and 1992-1994 two cohorts study)	[190]
OTHERS			
**Brazil**	12.40	From 13.07 to 12.40 (born in 1920-1979)	[197]
**Chile**			[198]
Indigenous	12.60	From 13.70 to 12.60 (born in 1920-1979)	
Non-indigenous	12.40	From 12.90 to 12.40 (born in 1920-1979)	

**Colombia**	12.70	From 14.30 to 12.70 (born in 1941-1989)	[199]
**Israel**	13.03	From 13.41 to 13.03 (born before 1970 and after 1978)	[200]
**Bangladesh**	13.54	From 12.86 to 13.54 (born before 1979 and after 1986)	[201]
**New Zealand**	12.90	n/a	[202]

*-median AAM

Table S2: Association between early age at menarche and in utero exposure in environmental hazards

Substances	Cohort	Association with AAM*	Difference in mean AAM, years	References
PBB	327 American females aged 5-24; exposed in utero to PBB dose 17.3pbb.	Association with early AAM was found	-1.1	[55]
DES	4429 American girls exposed to DES in utero	Borderline association with early AAM was found	-	[52]
DES	34 208 Puerto Rican and US females from Sister Study aged 35-59	Association with very early AAM (<10) was found	-	[169]
PCBs; DDE	213 American female offspring exposed to PCBs and DDE in utero	No association with PCBs exposure was found. For DDE, the association was non-significant	-	[61]
Polyfluoroalkyl chemicals	5756 female offspring of the ALSPAC prospective cohort	Association with changes in AAM was not found	-	[203]
Tobacco, alcohol	994 American female offspring aged 5, 9-11 and 15-17.	Association between early AAM was found in non-Caucasian offspring of smoking mothers; no association with alcohol exposure in utero.	-0.33 (for smoking only)	[65]

Maternal smoking	1556 American multi-ethnic female offspring. Retrospective cohort study.	Maternal smoking was associated with later AAM in the offspring of heavy smokers.	+0.31	[64]
Maternal smoking	262 American multi-ethnic women. Retrospective cohort study.	Association with later AAM was found	+0.44	[63]
Maternal smoking	34208 Puerto Rican and US females from Sister Study aged 35-59	Association with early AAM was found	-	[169]
Maternal smoking	2046 premenopausal and 1092 postmenopausal Japanese women	Association with early AAM was found for those daughters who were born after 1971	-0.31	[170]
Marihuana exposure	69 Canadian female offspring aged 13-16 of marihuana users. Retrospective cohort study	Association was not found	-	[204]
Smoking and alcohol		Association with alcohol was not found	-	[205]
Maternal smoking	34208 Puerto Rican and US females from Sister Study aged 35-59	Association with very early AAM (<10) was found	-	[169]

Maternal smoking	367 Danish girls aged 20-21, from Danish pregnancy cohort	Daughters who exposed to more than 10 cigarettes daily during first and second trimester of gestation had early AAM	-0.5	[69]

* considered significant at $p<0.05$

Table S3: Association between AAM and birth size

Factor and confounders	Cohorts	Association with AAM*	Difference in mean AAM, years	References
Birth weight, length, ponderal index, head circumference and subscapular skin-fold thickness	262 Norwegian adolescent girls. Prospective cohort study.	Low birth weight (less than 3200g, n=80) was associated with earlier AAM. 12.58 vs 13.25 years	-0.8	[71]
Birth weight, gestational age	273 Polish girls aged 17-21. Prospective cohort study.	Low birth weight (less than 2500, n=27) was associated with later AAM	n/a	[51]
Birth weight, length	156 Australian girls aged 8-15. Prospective cohort study	Association with AAM was not found. After adjusting BMI z-score association was found in girls who were lighter and longer at birth (12.0±0.3) comparing with girls who was short and heavy (13.0±0.3) and long and heavy (12.5±0.1). Less than 2500 n=5	From -0.5 up to -1.0	[75]
Birth weight	262 American multi-ethnic women born between 1959 and 1963. Retrospective cohort study.	Association of early AAM with higher birth weight was not clear. (OR =2.59, 95% CI: 0.79-8.53)	-	[73]

Birth weight	4000 British girls aged 8-14. Longitudinal observation study	No association was found	-	[74]
Birth weight and length	997 Filipino girls, aged 14-15 from Cebu Longitudinal Health and Nutrition Survey (CLHNS) Longitudinal cohort study.	Girls who were lighter (<2.95 kg) had later age at menarche (>14 years) Association for early AAM was found only after length adjusting. Longer and lighter at birth girls had earlier AAM	-	[78]
Birth weight	34208 Puerto Rican and US females from Sister Study aged 35-59	Lower birth weight (less than 2,500g) was associated with early AAM rRR 1.33 [1.08–1.63]	n/a	[169]
Birth weight	438 White, Asian or Polynesian girls aged 12-18 from Female Adolescent Maturation (FAM) study	Lower 1,17[0.69–2.0] and higher birth weight HR 1.01[0.49–2.07] has no association with early AAM	-	[37]
Birth weight and	187 Catalan girls with	Those who had low birth weight less than -2SD	-0.8	[206]

precocious pubarche	precocious pubarche, 6 months after menarche. Longitudinal cohort study	attained menarche earlier than 12 years		
	54 Catalan (North Spanish girls) aged 8-9. Longitudinal cohort study.	Group with mean birth weight 2500 (SDS 2.2±0.2) had early AAM than group where birth weight mean was 3100 (SDS 0.1±0.1)	-1.6	[207]
Birth weight	38 Italian girls aged 17.5-18.5	AAM was non-significantly lower in those who were SGA	-0.4	[208]
Birth weight	679 Swedish girls aged 18. Longitudinal cohort study.	AAM was non-significantly lower in those who were SGA	-0.4	[47]
Birth weight	92 American females who had very low weight birth. Longitudinal cohort study	There was no difference in AAM was found with those who had appropriate weight at birth	+0.1	[209]
Birth weight and length	4465 Norwegian girls aged 13-19 years.	Girls who were lighter at birth (25-75 percentile) more often earlier AAM	-0.8	[210]
	81606 British women aged 16-98, from Breakthrough generations study.	Association early AAM with lighter birth weight was significant: OR 0.31 (0.19, 0.43) per each 500g for univariate analysis, OR 1.24 (1.10, 1.37) for multivariate analysis	-	[15]
	2715 British girls from prospective birth cohort	No significant association was found 3.38±0.02 vs 3.36±0.02	-	[95]

	study			
	2058 British girls aged 15. Longitudinal study	After adjusting growth characteristics in infancy and childhood, association with early AAM was not found HR 0.96 (0.87-1.05)	-	[211]
BMI in early childhood	349 Australian adolescent girls aged 12-15 from The Western Australian Pregnancy Cohort (Raine) Study. Prospective longitudinal study.	Those who have low birth weight and high BMI at age 8, attained menarche earlier than girls who were heavier at birth but lighter at age 8. Median age and range 12.5 (9.4-14.4) vs 13.2 (11.0-14.2)	-0.7	[179]
BMI z-score in infancy and early childhood	305 adolescent girls from North Carolina Infant Feeding Study. Longitudinal prospective study.	Higher birth weight and greater weight gains over the four age intervals during infancy and early childhood were consistently associated with earlier menarche β= -0.06 (-0.10, -0.03)	-	[173]
	652 American girls aged 8-15 from NHANES cohort. Longitudinal study.	Association was not significant β –0.24(-0.60; 0,12) CI 95%, p 0.186	-0.24	[212]
	96493 French women aged 40-65 from E3N cohort. Longitudinal prospective	Women with a high birth weight had their menarche on average 1.5 months later than those with a low birth weight (P trend < .0001) β 1.51	-0.13	[16]

	study.	(0.87; 2.16) CI 95%.		
	1309 Turkish female students	Low birth weight was associated with late AAM. Median, range 14 (11-15) vs 13 (10-16), p 0.002	+1.0	[213]
Birth weight	2278 Asian American females with/without breast cancer. Case-control studies	Higher birth weight was associated with early AAM in both groups (p=0.016 and p=0,020).	n/a	[214]
Birth length, gestational age, BMI-for-age percentile, MET score, and mother's AAM	203 American female participants in the Dietary Intervention Study in Children (DISC)	unadjusted test for trend analyses, both quartile of birth weight and birth weight modeled as a continuous variable were positively associated with AAM (p < 0.01)	-0.51	[215]
Prepubertal body mass index, parental heights, parental ages, highest educational attainment of either parent, school grade point average of the partic-ipant at age 15–16 yr, mother's smoking dur-ing pregnancy, whether parents had divorced	65 Finnish females with very low birth weight and 92 control from Helsinki Study of Very Low Birth Weight Adults	There were no differences in reported AAM between very low birth weight subjects and controls	-	[216]

*-considered significant at p<0.05

Table S4: Postnatal exposure of environmental hazards and AAM

Substances	Subjects	Association with AAM*	Difference in mean AAM, years	References
Tobacco<br>Alcohol<br>Drugs, cannabis, hallucinogens, solvents, glues.	295,042 Canadian girls	No association was found between the AAM and any of the substance use outcomes	-	[79]
Passive (postnatally only) or<br><br>active (at time of menarche) tobacco smoke exposure	163 girls of multi-ethnic birth cohort who were highly exposed (more than 8 packs/year)<br>59 girls of the same cohort (California, USA)	Earlier AAM (-0.15 years) non-significant association<br><br>Later AAM (13.7 years), association was not found	-	[65]
Passive tobacco smoke exposure (postnatally only)	90 girls of multi-ethnic birth cohort (Baltimore, USA)	Later AAM, association was found	+0.55	[63]
Maternal smoking	26008 Polish girls	Maternal smoking was associated with early AAM	n/a	[80]

*-considered significant at $p<0.05$

Table S5: Association between body size, fat distribution and the AAM

Anthropometric characteristics	Cohorts	Association with AAM*	Difference in mean AAM, years	References
Weight<br>Relative weight<br>Weight velocity<br>Height<br>Height velocity	427 US Caucasian girls of age 9 in the NHLBI Growth and Health Study (NGHS) in 1980 and 67 US Caucasian girls<br>in the Berkeley Guidance Study (BGS) born in 1928-29	For NGHS cohort the strongest association with weight and relative weight.<br>For BGS cohort – skeletal maturation had the strongest association	n/a	[217]
Weight in<br>0-6 months<br>6-12 months<br>1-2 years<br>2-5 years	305 US multi-ethnical girls	Inverse association with higher birth weights and greater weight gains during any period of infancy and early childhood	-1 year for black girls	[173]
BMI	4,669 Danish girls	High prepubertal body mass index is associated with earlier<br>AAM	n/a	[180]
BMI	2145 US girls (NHANES	AAM had no significant influence on BMI	-	[218]

	cohort)	in adolescents		
BMI,<br>Height,<br>Waist circumference,<br>Percentage of body fat	6156 Chinese girls	High waist circumference is closely associated with AAM	n/a	[7]
Waist circumference,<br>Hip circumference,<br>Hip-waist ratio,<br>Triceps skin folder	693 US girls, NHANES multi-ethnic cohort	High lower body fat is more associated with AAM than upper and overall body fat	n/a	[90]
BMI	27878 15 years-olds girls in 34<br>countries and 18,101 11-year-olds girls in 29 countries (Europe and North America)	The association was found on the country and individual level	-0.5	[219]
BMI	776 girls from West Australian Pregnancy (Raine) Cohort Study	Girls with BMI above the median at age 8 had early menarche	n/a	[179]
BMI,<br>Percentage of body fat,<br>fat mass/height^2	107 German girls, DOrtmund Nutritional and Anthropometric	Body composition is not critical for puberty onset.<br>BMI and fat mass/height^2 reflect a tempo	-	[91]

	Longitudinally Designed (DONALD) Study	of later pubertal stages attainment		
BMI in 2, 9 and 19 months age	2715 UK girls	Rapid weight gain during 2-9 months was associated with earlier AAM.<br>There was no association between weight gain in 9-19 months and AAM.	n/a	[95]
Weight, waist circumference, and body composition by dual X-ray absorptiometry at age of 8 years	149 Australian girls, predominantly European descents	BMI z-score at age 8 years was associated with earlier AAM	n/a	[75]

*-considered significant at $p<0.05$

Table S6: Association between some alimentary products and AAM

**Alimentary products, compounds or supplementation**	**Cohorts**	**Association with AAM***	**Difference in mean AAM, years**	**References**
Animal and vegetable protein	DONALD study, 57 German girls	Animal protein intake in age 5-6 was associated with early AAM. Vegetable protein intake in age 3-4 and 5-6 resulted in delayed puberty.	-0.6	[103]
Animal and vegetable protein	Nepalese school girls	Vegetarian diet was associated with later AAM	+0.14	[194]
Dietary fibre, Cellulose, Animal fat	637 premenarcheal girls, Canada	High fibre and cellulose intake was associated with later AAM. High animal fat intake can result in earlier AAM	n/a	[105]
Protein, fat, carbohydrates	213 American girls, 10 years old	AAM was not associated with energy or non-energy intake of protein, fat and carbohydrates	-	[220]
Soybean products	2920 UK Caucasian girls from Avon Longitudinal Study of Parents and Children (ALSPAC)	Soy products in early infancy can increase the risk of early AAM	n/a	[107]

	cohort			
Soy formula	34208 Puerto Rican and US females from Sister Study aged 35-59	Soy formula was associated with very early AAM and with late AAM	-	[169]
Soy formula	338 American females, aged 20-34, retrospective cohort study	AAM was the similar as in those who were fed with cow milk formula	-	[221]
Isoflavones and fibre	119 German girls from DONALD cohort	Neither isoflavones nor dietary fibre had any significant association with later puberty events.	-	[108]
Animal protein	3298 pubertal girls from ALSPAC cohort	High animal protein (meat) intake in age 3-7 years had a strong association with early AAM	n/a	[104]
Vitamin D	3202 Colombian girls aged 5-12 years from Bogota School Children cohort.	Vitamin D deficiency was associated with earlier AAM	-0.8	[109]
Dairy products	1308 multi-ethnic girls aged 9-12 from NHANES cohort, USA	There is some evidence that greater milk intake could lead to early AAM	-	[106]
Dairy products	134 prepubertal Iranian girls aged 4-12	The risk of early AAM was higher in those who had higher milk intake	-	[222]
Nuts and seeds	777 Spanish girls, aged 8-16	Significant association with early AAM was found	-	[223]

*-considered significant at $p<0.05$

Table S7: Association between postnatal chemical hazards exposure and the AAM

Substances	Cohort	Association with AAM*	Difference in mean AAM, years	References
Persistent organochlorine pollutant	545 Swedish women, retrospective study	No association was found	-	[118]
PBBs	327 USA females aged 5-24	No association with exposure during breastfeeding	-	[55]
PCB, DDE, lead, mercury, mirex	138 Akwesasne Mohawk Nation girls aged 10-16.9, Canada	Association was found with exposure of lead in low dose combined with PCB congeners and earlier AAM	+0.88	[58]
Hair products (monoethylphtalate, methylparabene, oestrogen, placenta)	300 multi-ethnic women aged 18-77, retrospective study	Childhood hair oil and perm using associated with earlier AAM	n/a	[122]
Lead	1682 South African girls aged 13	High blood lead significantly associated with delayed AAM	n/a	[121]

DDT	466 Chinese female textile workers aged 20-34	DDT exposure was associated with earlier AAM	-1.11	[120]
Lead	2050 Polish girls aged 7-16 years	Increased lead level in girls' blood was associated with later AAM in 2007	+0.69	[189]
Dioxin	282 women from Sverso Women's Health Study, who was premenarcheal in 1976	There was no association with menarcheal onset with 10-fold increase in dioxin	-	[53]
2,5-DCP, parabens, bisphenol A, triclosan, benzophenone-3, total phthalates, and 2,4-DCP	440 American adolescent girls NHANES cohort. Cross-sectional study	Environmental phenols exposure was associated with early AAM. Parabens, bisphenol A, triclosan, benzophenone-3, total phthalates, and 2,4-DCP did not affect the AAM	-0.6	[224]
PCBs	792 Flemish girls aged 14-15	Exposure to PCBs was associated with later AAM	n/a	[225]
Polybrominated diphenyl ethers	271 American girls from NHANES cohort	High concentration of polybrominated diphenyl ethers in serum was associated with early AAM	-0.1	[182]
Perfluorocarbons	2931 American girls, aged 8-18	High serum concentrations of perfluorocarbons was associated with delay in AAM	+0.25-0.5	[226]

*-considered significant at $p<0.05$

Table S8: Association between certain psychological factors during childhood and adolescence and the AAM

Psychological factors	Cohort	Association with AAM*	Difference in mean AAM, years	References
Childhood hardships, sexual abuse	4,524 female aged 11-16 from National Child Development Study Cohort, UK. Prospective cohort study.	More than two hardships in early life had the strongest association with late menarche (AOR, 2.32; 95% CI, 1.12-4.80). Sexual abuse was most strongly associated with early menarche (AOR, 2.60; 95% CI, 1.40-4.81).	n/a	[138]
Early life stress, child maltreatment	100 multi-ethnic American girls aged 10-13. Prospective cohort study	Individuals who had experience of sexual abuse attain AAM 1 year earlier than non-abused	-1.0	[137]
Father absence	10,847 multi-ethnic US women, aged 15-44 Retrospective cohort study	Divorce/separation from birth till 5 years old predicted early AAM	n/a	[128]
Autistic-like traits	383 Caucasian girls, Australia from Raine cohort. Prospective cohort study	Autistic-like traits before age 2 years associated with later AAM	+0.35	[139]

Single parenting, family conflicts	523 Mexican-American girls aged 11-13.<br>Prospective longitudinal cohort study.	Conflict-prone family environment significantly associated with early AAM (before 11 years) (OR=2.22; 95%CI: 1.12, 4.40)	n/a	[129]
Father absence	161 girls from New Zealand	Younger sisters from disrupted families who were exposed to serious paternal dysfunction in early childhood attained menarche 11 months earlier than their sisters from disrupted families who were not exposed to such dysfunction.	-0.92	[130]
Father absence	708 French women aged 18-30<br>Retrospective cohort study	Significant association was found between father absence at age 5-15 with early AAM	-0.45	[131]
Early childhood stress	197 postmenarcheal Italian women aged 16-19	More stress in family life in childhood (birth to age 11), more parental marital unhappiness in childhood (birth to age 11), more conflict with mother in childhood (birth to age 11), more rejection from father in childhood (birth to age 11), less emotional closeness to mother in childhood (birth to age	n/a	[136]

		11), and more behavioural independence from mother or father in late childhood (age 8 to 11) were associated with earlier menarche		
Mother's harshness	433 White, 62 Black, and 31 Hispanic American females	Maternal harshness at ages of 54 months predicts early AAM	n/a	[227]
Family composition	1938 multi-ethnic college students, USA	Absence of a biological father, the presence of half- and step-brothers, and living in an urban environment were associated with earlier menarche.	-0.39	[132]
Family composition	273 Australian women aged 18-75. Cohort retrospective study.	Association between elder brothers and later AAM of their sister was found	+0.9	[228]
Father absence, sibship	5913 British women aged 16-44 from National Survey of Sexual Attitudes and Lifestyles 2000 (NATSAL 2000) Retrospective cohort study	Father absence associated with early AAM; number of younger siblings and older sisters can predict later AAM	n/a	[133]
Father absence	83 multi-ethnic American girls aged 11-14.	Early father absence was associated with early AAM	n/a	[134]

	Prospective cohort study.			
Childhood sexual abuse	2225 women under 65 from Otago Women's Health Child Sexual Abuse Survey, New Zealand. Retrospective cohort study.	Childhood sexual abuse was associated with early AAM (near 50% abused respondents)	n/a	[135]
Childhood adversities	1999 (for 20–24 year olds), 2000 (for 40–44 year olds), or 2001 (for 60–64 year olds) Australian women from PATH Through Life Project. Retrospective cohort study.	Adverse childhood experiences were associated with early AAM	-0.24	[127]
Autism spectrum conditions	35 women with autism spectrum conditions aged 18-58, USA. Cross-sectional survey.	Autistic conditions were associated with later AAM	+0.7	[140]
Autism, cerebral palsy,	44 adolescents aged less	No significant association was found for all		[141]

Down syndrome	than 21 with such conditions: Down syndrome 13, autism 14, cerebral palsy 17.	groups	-	
Anorexia nervosa	33 French Caucasian girls with anorexia nervosa. Retrospective cohort study	Females with anorexia nervosa attained menarche significantly later (15.4 vs. 13.2 years)	+2.2	[142]

*-considered significant at $p<0.05$

Table S9: Relationship between socioeconomic conditions and the AAM

Socio-economic variables	Cohort	Association with AAM*	References
Living in the urban or rural area	15388 Chinese girls aged 6-19. Retrospective cross-sectional study	Urban girls attaint AAM earlier than rural ones (12.28 vs. 12.50)	[155]
Living in the urban or rural area	1100 adolescent girls from Central India. Cross-sectional descriptive study	Mean ages of menarche were 13.51±1,04 years and 13.67±0.8 years for urban and rural areas respectively	[149]
Living in the urban or rural area	708 Nigerian adolescent girls. Cross-sectional study.	There was no significant difference in the age at menarche between the rural and urban school girls (15.32 years and 15.20 years)	[150]
Living in the urban or rural area	92 767 girls in 2005 and 85 433 girls in 1995 aged 9-18 from National Physical Fitness and Health Surveillance in China. Cross-sectional study.	AAMs were 12.60 and 12.92 years for urban and rural girls, respectively. No significant difference	[160]
Living in the urban or rural area	1611 Chinese women aged 19-45	Significant difference in AAM between women from city and country was found (14.2±1.5 vs. 14.6±1.5)	[156]
Living in the urban or rural area	715 adolescent females from Bengali-speaking Hindu ethnic group. Cross-sectional study	The AAM was significantly lower in the urban girls (11.42 ± 1.03 vs. 12.10 ± 1.15)	[151]
Living in the urban or	612 Ethiopian girls aged 14-19. Cross-	The mean AAM for rural female adolescents	[152]

rural area	sectional study	was significantly higher than for urban ones (15.1 vs. 14.4)	
Living in the urban or rural area	2090 Iranian adolescent girls aged 14-17. Cross-sectional study.	There wasn't significant difference between urban and rural resident	[153]
Living in the urban or rural area	410 South Korean girls, aged 6, 9, 13 and 15 years. Cross-sectional study.	Urban girls had menarche onset significantly earlier (12.6 vs. 13.0 years)	[229]
Living in the urban or rural area	214 Chinese girls, aged 13-16 years. Cross-sectional study.	Urban females AAM was significantly earlier than rural ones (12.8 vs. 13.2 years)	[157]
Living in the urban or rural area	911 Cameroonian girls aged 9-19, cross-sectional study	Urban residence was associated with earlier AAM (13.18 vs. 14.27)	[154]
Socioeconomic status	407 Chilean girls (indigenous and non-indigenous)	Low SES was associated with delayed menarche (5.4 months later), regardless of nutritional status or mother's AAM	[158]
Living in the urban or rural area; Socioeconomic status	720 Ghanaian females, aged 7-17. Cross-sectional study.	Early AAM was associated with urban residence (12.37±1.48 vs 13.41±2.25). High SES was a predictor of early AAM	[185]
Living in the urban or rural area	1938 multi-ethnic college students, USA	Urban residence was associated with earlier AAM (12.37 vs. 12.84)	[132]
Socioeconomic status	4868 Turkish girls aged 6-18. Cross-sectional study.	Affiliation to upper socioeconomic class was associated with earlier AAM (12,60 vs. 12,83)	[159]
Parents' educational	608 Polish rural girls aged 9-18	AAM was associated with parents' educational level	[230]

level; source of income; household appliances; the number of children in a family		and the number of children in family	
Socioeconomic status	262 multi-ethnic American females aged 38-43. Retrospective cohort study	Low socioeconomic status at age 7 is positively associated with early AAM	[163]
Household income; parental education	1,091 black and 986 white girls aged 9-10 from USA	White girls from low-income families and black girls from high-income families had more risk of early AAM. Parental education had no association with AAM	[164]
Household income, education, place of residence and employment status province of residence, immigration status, ethnicity, place of birth, language first learned at home and family type	1403 Canadian girls aged 14-17. Prospective cohort study.	Household income and family type were associated with AAM	[231]
Socioeconomic level	3783 Italian high school girl. Cohort	No association was found	[20]

	prospective study		
Parental occupational and educational level	1017 Turkish female adolescents aged 14-18. Prospective cohort study	No significant association was found	[161]

*-considered significant at $p<0.05$
